# Anaesthetic efficacy of Aqui-S, Benzoak, and MS-222 on lumpfish (*Cyclopterus lumpus*) fries. Impact from temperature, salinity, and fasting

**DOI:** 10.1371/journal.pone.0211080

**Published:** 2019-01-22

**Authors:** Julianne Valla Jacobsen, Klemet Steen, Kjell J. Nilssen

**Affiliations:** 1 Lerøy Midt AS, Sandstad, Norway; 2 Lerøy Seafood Group ASA, Bergen, Norway; 3 Department of Biology, Faculty of Science and Technology, Norwegian University of Science and Technology, Trondheim, Norway; Institute of Marine Research, NORWAY

## Abstract

Large numbers of lumpfish are produced for the Norwegian salmon industry and are used to combat sea lice infestations. Periodically high mortality of farmed lumpfish demonstrates the need to improve farming conditions and animal welfare. As part of such efforts, the present work tested the efficacy of three anaesthetic chemicals on lumpfish fries (average weight of 0.97 g). The anaesthetic impact from salinity (15 ppt–18 ppt), temperature (12°C versus 7 and 18°C), and fasting conditions (three days) was also examined. Surgical anaesthesia was induced within 3 to 5 min (preferred time) at concentrations of 18 mg/L (Aqui-S), 37.5 mg/L (Benzoak), and 60 mg/L (buffered MS-222). Safety margins were regarded as low when using Aqui-S; therefore, this chemical was not considered suitable for prolonged exposures. The lumpfish made a rapid recovery from both Benzoak and MS-222 even after 20 min of exposure. A 6°C increase in exposure temperature (reaching 18°C) was found to delay or inhibit recovery. The effect of a 5°C decrease (down to 7°C) significantly reduced induction time for MS-222 and was insignificant for Aqui-S, while it prolonged Benzoak induction time significantly and gave a longer recovery period. Fasting resulted in 70% recovery after 20 min of Aqui-S exposure compared to 0% in fed fish but had only minor effects on Benzoak and MS-222. Use of brackish water (15 ppt–18 ppt) gave 20% recovery from Aqui-S and significantly shorter recovery time from MS-222 exposure, while the effects on Benzoak were insignificant.

## Introduction

The Norwegian farming of Atlantic salmon (*Salmo salar*) has reached a yearly production of 1.2 million tons, representing a value of 7.2 billion USD [1]. Salmon infestation by an ectoparasitic lice (*Lepeophtheirus salmonis*) has become a major challenge to this important industry. These lice result in impaired fish welfare, reduced fish survival, and high costs since delousing activities amounted to 309.6 million USD in 2016 (including indirect costs) [2].

The use of delousing chemicals (such as azamethiphos, pyrethroids, emamectin benzoates, and hydrogen peroxide) are not acceptable anymore as they negatively impact the local animal environment, e.g. by influencing moulting in crustaceans [3]. In addition, these delousing chemicals have lost their potency due to the lice achieving resistance [4, 5]. Recently developed mechanical delousing strategies can be effective with respect to lice removal but have also been shown to compromise fish welfare and survival [6]. Currently, biological cleaning by using lumpfish (*Cyclopterus lumpus*) has shown great promise. Thus, several Norwegian companies produced and released ~15.6 million lumpfish fingerlings at sea pens in 2016 [7]. Furthermore, an estimated yearly requirement exceeding 50 million is now making the lumpfish the second most important farmed fish in Norway.

However, challenges in lumpfish farming, including uneven growth rate and periodically high mortalities [8, 9], indicate that the Norwegian lumpfish farming requires improvements. As a starting point, it would be necessary to limit the lumpfish’s stress exposure related to transport and handling. One way is to practise sedation or anaesthesia prior to and during farming procedures.

The common way to immobilize fish is by dissolving chemicals in water for absorption over the gills (inhalation), and the stage of anaesthesia is normally assessed by behaviour and activity of the fish [10]. The effect of drugs can vary within and between species with biological factors, including sex, age, life stage, growth, health, stress, and also with abiotic factors such as salinity, pH, oxygen, and temperature [10–12]. An anaesthetic agent for use in aquaculture should have a short induction time (3–5 min) and recovery within 10 min (modified from [13]). Concentrations used for treatment should also have low toxicity, thus providing a satisfactory safety margin, and in addition, should pose low risk for humans and the environment. Currently, MS-222, Benzoak, and Aqui-S have become the sedatives most commonly used in Norwegian salmon farming. Isoeugenol (2-methoxy-4-prop-1-enyl-phenol), the active agent in Aqui-S, is found in clove oil and functions by inhibiting voltage dependent ion-channels, potentiation of gamma amino butyric acid _A_ (GABA_A_) receptors, and inhibition of N-methyl-D-aspartate (NMDA) receptors (reviewed in [10]). The substance is harmless to humans, and the withdrawal period after use is only two degree days [14]. The active agents in MS-222 (metacaine, ethyl 3-aminobenzoate) and Benzoak (benzocaine, ethyl 4-aminobenzoate) are closely related and have the same mode of action via blocking sodium channels leading to blockage of most neurons and muscle cells [15, 16]. The more soluble MS-222 will reduce seawater’s pH; hence, this agent can function as an irritant and also affect blood chemistry. Buffering the solution (with sodium bicarbonate for example) is therefore necessary to counteract this effect.

Skår and co-workers [17] have recently published a well-documented protocol for anaesthesia of medium and large sized lumpfish. However, protocol for anaesthesia of the small lumpfish fries (~1 g) at the ongrowth facilities is still not available. Accordingly, the purpose of the present work was to supply information and protocols for the efficacy of Aqui-S, Benzoak, and MS-222 for anaesthesia of lumpfish fries. Because change in wind- or sea currents can alter seawater temperature, we wanted to study the significance of un-acclimated temperature changes on anaesthetic effect. Furthermore, we examined the effect of salinity and nutritional status on these agents.

## Materials and methods

The performed experiments were approved by the Norwegian Animal Research Authority (Mattilsynet)–identification number FOTS ID 11840. Laboratory protocol: http://dx.doi.org/10.17504/protocols.io.wg2fbye

### Animal rearing conditions and location

Lumpfish fries (0.97 ± 0.45 g) were supplied from a lumpfish facility on growth (Lerøy Midt AS), located in Stokksund at the coast of Sør-Trøndelag county in Norway. The lumpfish were transported by car in a plastic container with oxygenated seawater to the university campus (Realfagsbygget at NTNU) in Trondheim. Upon arrival, the fish were carefully transferred to an aerated seawater (12°C) holding aquaria (~400 L). Food pellets (Gemma Diamond, 0.8 mm, Skretting AS) were supplied ad libitum and a simulated natural photoperiod LD 14:10 was maintained throughout the experimental period.

### Anaesthetics

Benzocaine (Benzoak vet, 200 mg/ml, EuroPharma, Leknes, Norway), metacaine (Tricaine methanesulphonate 1000 mg/g, Sigma Aldrich Co., St. Louis, USA, which is also named Finquel, or MS-222), and isoeugenol (Aqui-S, 540 mg/ml, Scan Aqua, Norway) were used in this study. Liquid Benzoak was stirred into seawater (12.5, 25, 37.5, 50, 100 mg/L), metacaine powder and the corresponding weight of sodium bicarbonate (Na_2_CO_3_) were dissolved in seawater at concentrations of 25, 38, 44, 60, 75, 100, and 150 mg/L. Isoeugenol (540 mg/ml) was diluted in ~5 mL heated (30°C) water prior to being added to the seawater to yield 6, 12.5, 18, 25, and 50 mg/L. The selected chemical concentrations were based on results from tests done prior to the experiments.

### Induction; immersion anaesthesia

Immersion anaesthesia was performed by transferring individual lumpfish fries from the holding tank into an aerated transparent box (5L) containing pre-mixed chemicals. The lumpfish fries displayed a behaviour different from other species during the preliminary induction tests. A pilot study of lumpfish behaviour development during anaesthetic exposure was carried out prior to the immersion experiments. The visual changes from natural swimming included disorientation, loss of equilibrium (often swimming sideways or upside down), no swimming (falling to the tank bottom), reduced fin movements, and finally inactivity with reduced respiration. The fish were considered to be surgically anaesthetized (known as stage 3b) at the last behavioural stage. The time to induce stage 3b was measured for 10 lumpfish. Observations were terminated if the anaesthesia was not reached within 10 min (600 sec), while the chemical concentrations inducing stage 3b within ~3 to 5 min were regarded as the Selected Best (SB) concentrations.

### Recovery—Therapeutic window

To investigate the safety margin of the anaesthetics (therapeutic window) batches of 10 lumpfish were exposed to the SB anaesthetic concentration for 5, 10, and 20 min. Thereafter, the fries were transferred to individual containers (~1 L) holding only seawater, and the time to full recovery was measured. The awakening behaviour for the lumpfish fries also diverged from that previously known for other fish species. After transfer to the recovery box, their activity changes included initial fin movements, increased gill movements, disoriented (sideways) swimming, swimming upright (but erratic), and normal swimming. Thus, the measured recovery corresponds to the time after transfer to standard seawater from unresponsiveness to displaying a normal swimming activity. Observations were terminated (T) if the fish had not recovered within 15 min (900 sec).

### Anaesthesia in brackish water, after fasting, or at different temperatures

Recordings of induction and recovery times (after 20 min exposure) were repeated with SB concentrations to assess the impact of salinity (15–18 ppt), nutritional status (3 days fasting), and temperature (7, 12, or 18 °C) on anaesthesia of the lumpfish fries. Brackish water (15 ppt–18 ppt) was made by mixing seawater and freshwater, and recovery after anaesthesia was obtained in seawater. Lumpfish used to assess nutritional status were kept fasting in separate aerated holding tanks for three days prior to performing the experiment. Acclimation was not allowed before temperature trials; lumpfish were transferred directly from the holding tank (12°C) to experimental boxes containing pre-mixed anaesthetics at a lower (7°C) or at a higher (18°C) temperature. Recovery was performed in seawater kept at the corresponding temperature.

### Statistical analysis

Graphing and statistical analysis were conducted using SigmaPlot 13 (Systat Software Inc., Uk). The induction and recovery times of anaesthetic chemical concentrations and comparison between acclimated and alternating conditions were analysed with the one-way analysis of variance (ANOVA) to test for differences. When the normality (Shapiro-Wilk) or equal variance test (Brown-Forsythe) failed, ANOVA on Ranks (Kruskal-Wallis ANOVA on ranks) was used. When significant differences were detected, a multiple comparison test (Tukey test or Dunn´s Method) was conducted to isolate the differing groups. Values are given as mean ± standard deviation (SD). Statistical significance was set to P <0.05.

## Results

### Full anaesthesia and selected best (SB) induction concentrations

**Fig 1:**
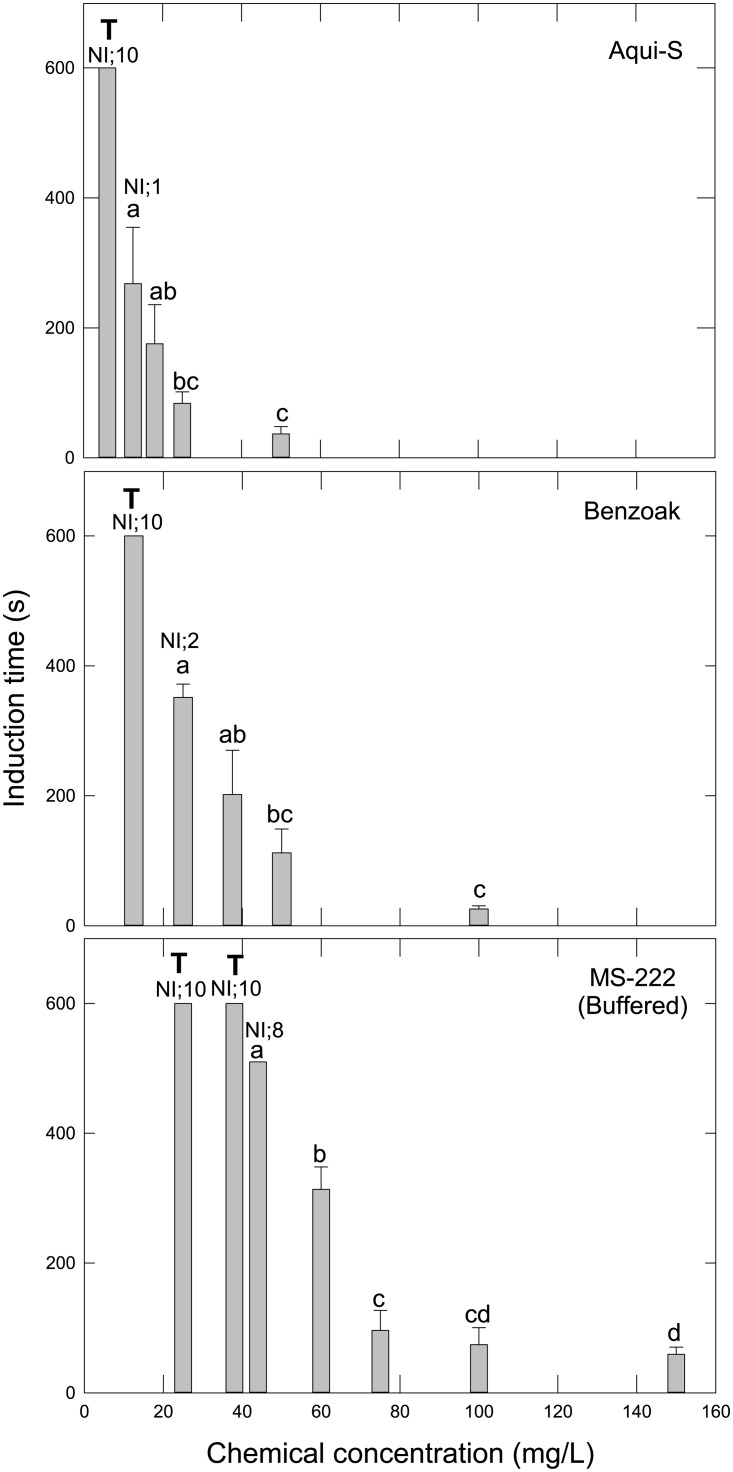
Anaesthetic induction. Time to surgical anaesthesia (stage 3b) for lumpfish fries (*Cyclopterus lumpus*) subjected to various Aqui-S, Benzoak, and MS-222 concentrations. Values are mean + SD, N = 10 (except MS-222 100mg/L; N = 20). If stage 3b was not reached within 600 sec, the experiment was terminated (T). NI; number of fish Not Induced to stage 3b. Different letters above bars indicate statistical differences.

While all of the tested chemical concentrations affected fish behaviour, the lowest dosages did not induce full anaesthesia (within 600 sec). A negative relationship was found between induction time and increasing chemical concentrations (Fig 1). Concentrations required to induce stage 3b within ~3 to 5 min were 18 mg/L, 37.5 mg/L, and 60 mg/L for Aqui-S, Benzoak, and MS-222, respectively. These concentrations were therefore named the SB concentrations and were used to test the lumpfish therapeutic window (recovery) at increasing dosages.

### Recovery—After SB induction

**Fig 2:**
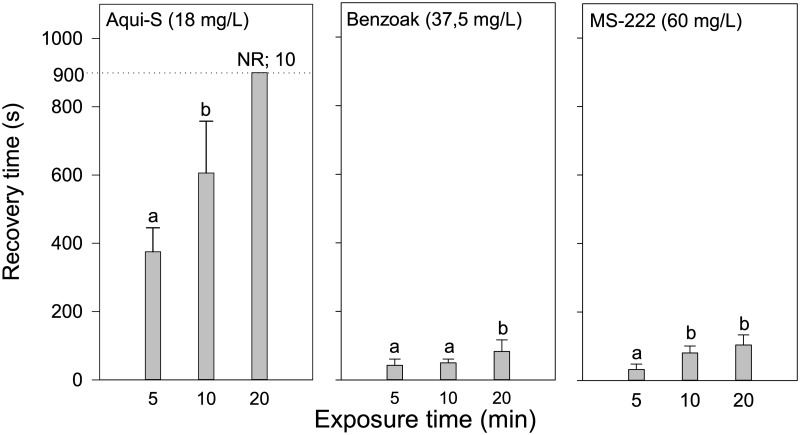
Recovery. Time to recovery for lumpfish fries (*Cyclopterus lumpus*) after exposure to Selected Best (SB) concentrations of Aqui-S, Benzoak, and MS-222. Values are mean + SD. NR; number of fish Not Recovered within 900 sec. Different letters above bars indicate statistical differences.

Using the SB concentrations, 5 min of exposure gave a recovery within 1 min for Benzoak and MS-222, while the corresponding average recovery time after Aqui-S exposure was 6 min and 15 sec (Fig 2). Increasing exposure time to 10 min resulted in only a minor increase in recovery times from Benzoak and MS-222, while an increase to 10 min resulted after Aqui-S treatment. Recoveries from Benzoak and MS-222 were still rapid after 20 min of exposure (being on average 83 and 104 sec, respectively), while no fish recovered within 15 min (900 sec) after 20 mins of Aqui-S exposure.

### Impact on anaesthetic induction from salinity, temperature, and fasting

**Fig 3:**
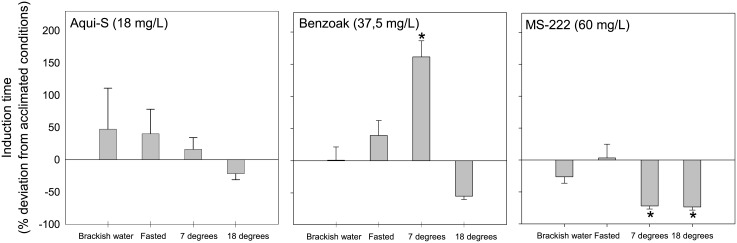
Induction under altered conditions. Deviation in induction time of lumpfish fries (*Cyclopterus lumpus*) to surgical anaesthesia (stage 3b) by SB concentrations of Aqui-S, Benzoak, and MS-222 when fries were exposed to these agents in brackish water (15 ppt–18 ppt), when fasted for three days, or at temperature lowered by 5 °C to 7°C or increased by 6°C to 18°C. Values are mean ± SD. N = 10. Asterisks indicate statistical differences from acclimated condition.

Reducing salinity from 34 ppt (seawater) to 15 ppt–18 ppt produced only a marginal (<1%) impact on Benzoak induction time (Fig 3), while the time to 3b stage decreased by 26% for MS-222 and increased by 47% for Aqui-S exposure. Conducting the experiment with the 3-day fasted lumpfish did not change the MS-222 induction time but prolonged the induction time by 39% and 40% for Benzoak and Aqui-S, respectively. Lowering the temperature from 12 to 7°C delayed the Aqui-S induction by 16% and Benzoak induction by 161% (P = 0.002). On the other hand, the time required to surgical anaesthesia in MS-222 decreased by 72% (P <0.001). Increasing the temperature from 12 to 18°C reduced the time to lumpfish surgical anaesthesia by 22%, 55%, and 74% in Aqui-S, Benzoak, and MS-222, respectively (P <0.001).

### Impact from salinity, temperature, and fasting on recovery from anaesthesia

**Fig 4:**
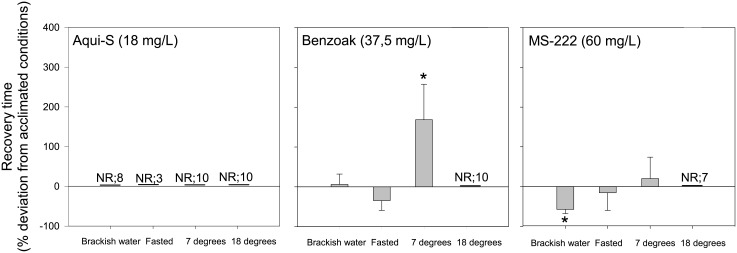
Recovery at altered conditions. Deviation in recovery time of lumpfish fries (*Cyclopterus lumpus*) after 20 min exposure to SB concentrations of Aqui-S, Benzoak, and MS-222 when fish were exposed to these agents in brackish water (15 ppt–18 ppt), when fasted for three days, or at temperature lowered by 5 °C to 7°C or increased by 6°C to 18°C. Values are mean ± SD. N = 10. NR; number of fish Not Recovered within 900 sec. Asterisks indicate statistical differences from acclimated condition.

Fig 4 illustrates the effects from brackish water, fasting, and temperature changes on recovery time after 20 min exposure to different SB concentrations. Reduced salinity caused no effect on recovery time from exposure in Benzoak. the MS-222 exposure group required only half the original (SB) recovery time (P = 0.045), while eight of the fries did not recover from exposure to Aqui-S in brackish water. Lumpfish fries that had been fasted for three days displayed a decreased recovery time after all chemical exposures. (Three fish did not recover from the Aqui-S treatment). After 20 min exposure to SB concentration at the lowest test temperature (7°C), the recovery time increased with 20% for MS-222, 168% for Benzoak (P = 0.003), while the fish anaesthetized with Aqui-S were alive but had not recovered after 15 min in seawater. Increasing the temperature to 18°C caused only three fish to recover from MS-222, while none of the fish anaesthetized with Aqui-S or Benzoak recovered within the 15 min observation period.

## Discussion

### Salmon bio and production policies

Using lumpfish as a live bio-cleaner of salmon lice has demonstrated potential and received praise from consumers and environmentalists. Accordingly, the salmon farming industry has rapidly made large investments and initiated lumpfish production even though most of its physiology and responses to rearing conditions still are unknown. The current experience with respect to irregular mortality occurrence also demands solutions for improving farming conditions and lumpfish welfare. An immediate improvement would be to reduce the handling-related stress load to which lumpfish are subject by implementing use of anaesthetic chemicals when performing necessary rearing procedures. Low dosages, which sedate the fish, might be suitable for some events, while more invasive handling may require full anaesthesia. It follows that protocols for suitable chemicals to sedate or anaesthetize lumpfish fries currently should have a high priority within the salmon industry.

### Monitoring depth of anaesthesia

Zahl and co-workers [18] have given an overview of fish behavioural stages exhibited during exposure to anaesthetics. Their descriptions, however, did not fit with observed lumpfish responses, and alternative criteria for assessing anaesthesia-related lumpfish responses have been accordingly tailored by Skår and co-workers [17]. Thus, following an excitatory stage, sedated lumpfish were reported to display reduced activity and loss of equilibrium. At the beginning of stage III, operculum movements were weak, fin movements had stopped, and there were no swimming activities. Normally, one would verify this anaesthetic level by demonstrating no response to pinching of the fish tail. This test was not found applicable to the lumpfish [17]. Instead, it was recommended to test the responsivity of this species by pinching its lower lips as this regularly releases a bite reflex in non-anaesthetized lumpfish. Clearly, we should adopt the same procedure in our studies. The small size (0.97 g) of the lumpfish fries did not, however, allow for pinching the lips to provoke behavioural reactions. Instead, we had to consider the lumpfish fries to be in an anaesthetized state (plane III-1) when they reached a non-moving, slow breathing bottom lying position after a period of reduced and disoriented activity. In most cases, this anaesthetic level would be sufficient to block handling related stress release. Also, in the absence of applicable behavioural tests, the results from recovery (therapeutic window) studies should prevent this from becoming a lethal anaesthetic condition.

### Anaesthetic induction—Chemical efficacy

The inductive efficiency of chemical drugs may be determined by testing the dosage (concentration x time) necessary to anaesthetize the animal. Our study revealed the chemical potency to be in the order of Aqui-S > Benzoak > MS-222 with observed effects on lumpfish behaviour at concentrations of 12.5 mg/L, 25 mg/L, and 50 mg/L, respectively. Increasing the chemical concentration also decreased the time to anaesthesia induction in the fries, which is in accordance with results from similar tests with other species including cod (*Gadus morhua*) [19], Atlantic salmon [20], and European sea bass (*Dicentrarchus labrax*) [21]. Since these drugs introduce anaesthetic conditions more rapidly at higher concentrations, it is reasonable to suggest that osmotic pressure and simple diffusion are responsible for the main uptake of anaesthetics over the lumpfish gills. Furthermore, at a concentration of 18.5 mg/L (Aqui-S), 37.5 mg/L (Benzoak), or 60 mg/L (MS-222), the lumpfish fries reared at 12°C reached anaesthetic stage III within ~3 to 5 minutes of chemical exposure.

Of the SB concentrations, our results for Aqui-S exposure of lumpfish fries (0.97 g) correspond with the findings of Skår [17] that were conducted at similar temperatures on larger lumpfish (10–20 g). Our findings, however, are lower when compared to concentrations used to induce surgical anaesthesia in Atlantic salmon (≥ 30 mg/L [20]) and induction by clove oil (comparable to Aqui-S) in juvenile European sea bass (30–40 mg/L [21]), and juvenile gilthead sea bream (*Sparus aurata*, 40–55 mg/L [21]).

Lumpfish SB concentration of Benzoak (37.5 mg/L), which was approximately only one third of the one (100 mg/L) recommended for 10 to 20 g lumpfish by Skår and co-workers [17], clearly demonstrate the importance of anaesthetic protocols that are developed based on body size. On the other hand, our Benzoak results seem to match those given for species such as Atlantic cod (40 mg/L [19], 25–35 mg/L [22]), Atlantic halibut (*Hippoglossus hippoglossus*) (40 mg/L [18]), and Atlantic salmon (≥ 30 mg/L [20]). The resulting lumpfish fry MS-222 SB concentration (60 mg/L) was again lower than (100 mg/L) given for larger (10–20 g) lumpfish [17], but more in agreement with that given for Atlantic cod (75 mg/L [19], 55–60 mg/L [22]).

### Recovery from anaesthesia—Therapeutic window

When using anaesthetic drugs, it is crucial that the fish are able to recover rapidly afterwards. In our study, lumpfish recovery after 5 min of SB Aqui-S (18mg/L) exposure was more than 6 min and increased with exposure time. Such delayed recovery after the use of Aqui-S and clove oil has also been reported in other fish, including red pacu (*Piaractus brachypomus*, [23]), European sea bass and guilted sea bream [21]. This prolonged recovery could be related to anaesthetic drug deposition in adipose tissue resulting from the active agent’s lipophilic characteristics that require longer clearance times [24]. Reduced opercular ventilation during clove oil anaesthesia has also been expressed [21]. Lumpfish anaesthetized with Aqui-S seem to have respiratory problems as they come to the surface gasping for air during recovery, indicating anaesthesia-induced hypoxia. It is therefore our opinion that Aqui-S cannot be recommended for use on lumpfish, which also agrees with results in a study by Skår and co-workers [17] concerning large lumpfish. The therapeutic window was much wider when the lumpfish were subject to SB concentrations of Benzoak (37.5 mg/L) or MS-222 (60 mg/L).

### Use of anaesthetics in brackish water

Anaesthesia at lower salinity (15 ppt–18 ppt) did not cause any remarkable changes in induction or recovery time of SB anaesthetic concentrations. Immobilizing small lumpfish in seawater may affect their osmoregulatory abilities and cause an increase in blood plasma ionic content. Our results indicate that lumpfish fries may benefit from anaesthesia in brackish water. The next step should therefore be to test blood parameters (such as osmolality or chloride concentration) in fish anaesthetized in seawater versus brackish water to uncover eventual blood plasma differences due to salinity during anaesthetic procedures.

### Effect of fasting on anaesthetic efficacy

The nutritional status of an animal may affect its physiological responses to environmental challenges and could thus possibly have an impact on anaesthetic efficacy. Thus, it has been reported that food restriction can lower the metabolic rate and hence, the ventilation rate [25], which in turn would reduce uptake of chemicals. Anaesthetic responses of lumpfish fries restricted from feed for three days did, however, demonstrate only minor changes in induction and recovery times for Benzoak and MS-222. The results, therefore, indicate that anaesthesia of fasted lumpfish can be performed, which is positive considering that feed restriction often is part of the procedure before handling and transport of farmed fish. When testing fasted lumpfish fries with SB Aqui-S concentration 70% of the lumpfish recovered, compared to zero when fed. Currently, we do not have any explanation for this observation.

### Anaesthetic efficacy at non-rearing temperature

Performing anaesthesia at six degrees above the acclimated temperature demonstrated that the SB concentrations needed less time to induce surgical anaesthesia. This reduction is probably related to increased ventilation and gill blood perfusion rates to compensate for increased metabolic cost at lower seawater oxygen tension levels. The significance of these changes is demonstrated by reduction of the therapeutic window (lower recovery rate).

If SB chemical exposure was performed at five degrees below rearing temperature, a less uniform induction time result was obtained. While only a small increase was found for Aqui-S exposure, the time increased (by 161%) and decreased (by 72%) for Benzoak and MS-222, respectively. The corresponding results for recovery (after 20 min of exposure at this lower temperature) indicated that fish did not recover with Aqui-S, showed a significantly delayed recovery for Benzoak and only a minor recovery delay for MS-222.

A change in induction time and therapeutic window (recovery) was documented for pre-stressed Atlantic cod [22]. The lumpfish subjected to anaesthesia at non-acclimatized temperatures could possibly have experienced stress due to the abrupt temperature change. Catecholamines (CA), which are rapidly released when the stress reaction is induced, would have impacted the ventilation rate and blood flow through the gills [26]. CAs could hence increase the uptake of drugs and influence both induction and recovery times. On the other hand, cortisol-induced effects are less likely due to its slow release. Irrespective of physiological cause, our data demonstrate that use of these anaesthetic agents should be controlled and performed at normal rearing (acclimated) temperatures.

## Conclusion

The results from our studies suggest that both Benzoak (37.5 mg/L) and MS-222 (60 mg/L) gave satisfactory anaesthesia of lumpfish fries, had acceptable induction and short recovery times, and good safety margins. Acceptable Aqui-S anaesthesia resulted in a recovery time that was too long; thus, this chemical cannot be recommended for anaesthetic purposes in lumpfish fries. Reduced salinity and fasting had little influence on anaesthesia, but precautions should be taken to perform anaesthesia at the temperatures to which the lumpfish fry have acclimated. In order to optimize the anaesthetic protocol, further studies should include blood chemistry during- and following anaesthesia.

## Supporting information

S1 File Results. (DOCX)

S2 File Statistics. (DOCX)
