# Supplementary material for: Anaesthetic efficacy of Aqui-S, Benzoak, and MS-222 on lumpfish (Cyclopterus lumpus) fries. Impact from temperature, salinity, and fasting
Source: PLoS One. 2019 Jan 22;14(1):e0211080. doi: 10.1371/journal.pone.0211080 (PMC6342319; doi:10.1371/journal.pone.0211080)
Supplement: S1 File — (DOCX) [file pone.0211080.s001.docx]

Table S1. Induction time at acclimated conditions (12 ^o^C, seawater)

| Anesthetic chemical | Concentration (mg/L) | Induction time (average time (seconds) ± SD).  NI=Not Induced. |
| --- | --- | --- |
| Aqui-S | 6 | NI; 10 |
|  | 12.5 | 331.1 ± 215.9 |
|  | 18 | 175.4 ± 60.3 |
|  | 25 | 83.7 ± 17.8 |
|  | 50 | 36.8 ± 11.2 |
| Benzoak | 12.5 | NI; 10 |
|  | 25 | NI;1  432.3 ± 184.5 |
|  | 37.5 | 202.0 ± 68.0 |
|  | 50 | 112.2 ± 36.7 |
|  | 100 | 25.8 ± 4.8 |
| MS-222 | 25 | NI; 10 |
|  | 38 | NI; 10 |
|  | 44 | 766.7 ± 160.8 |
|  | 60 | 313.5 ± 34.4 |
|  | 75 | 96.2 ± 30.7 |
|  | 100 | 74.1 ± 26.3 |
|  | 150 | 59.4 ± 11.0 |

Table S2. Recovery time at acclimated conditions (12 ^o^C, seawater)

| Anesthetic chemical | Exposure time (min) | Recovery time (average time (seconds) ± SD).  NR=Not Recovered. |
| --- | --- | --- |
| Aqui-S  (18 mg/L) | 5 | 375.1 ± 70.3 |
|  | 10 | 606.2 ± 151.5 |
|  | 20 | NR; 8  1172.1 ± 62.8 |
| Benzoak  (37.5 mg/L) | 5 | 42.8 ± 18.2 |
|  | 10 | 49.9 ± 11.3 |
|  | 20 | 83.1 ± 33.8 |
| MS-222  (60 mg/L) | 5 | 32.0 ± 16.1 |
|  | 10 | 80.6 ± 20.2 |
|  | 20 | 103.8 ± 29.3 |

Table S3. Induction time and recovery time at various conditions and deviation from control.

| Anesthetic chemical | Condition | Induction time | | Recovery time after 20 minutes exposure | |
| --- | --- | --- | --- | --- | --- |
|  |  | Average time (seconds) ± SD. | Deviation from control (%) | Average time (seconds) ± SD.  NR=Not Recovered. | Deviation from control (%) |
| Aqui-S  (18 mg/L) | Control  (12 ^o^C, seawater) | 175.4 ± 60.3 |  | NR; 10 |  |
|  | Brackish water | 258.3 ± 111.5 | 47.26 | NR; 8  604.0 |  |
|  | Fasted | 245.8 ± 66.7 | 40.14 | NR; 3  691.4 ± 137.6 |  |
|  | 7^o^C | 203.6 ± 31.8 | 16.08 | NR; 10 |  |
|  | 18^o^C | 137.3 ± 16.5 | -21.72 | NR; 10 |  |
| Benzoak  (37.5 mg/L) | Control  (12 ^o^C, seawater) | 202.0 ± 68.0 |  | 83.1 ± 33.8 |  |
|  | Brackish water | 203.7 ± 41.6 | 0.84 | 88.1 ± 21.6 | 6.02 |
|  | Fasted | 281.3 ± 47.0 | 39.26 | 54.4 ± 20.4 | -34.54 |
|  | 7^o^C | 528.0 ± 50.6 | 161.39 | 223 ± 74.1 | 168.35 |
|  | 18^o^C | 90.8 ± 10.1 | -55.05 | NR; 10 |  |
| MS-222  (60 mg/L) | Control  (12 ^o^C, seawater) | 313.5 ± 34.4 |  | 103.8 ± 29.3 |  |
|  | Brackish water | 230.9 ± 31.5 | -26.35 | 44.3 ± 10.9 | -57.32 |
|  | Fasted | 323.8 ± 67.1 | 3.28 | 87.7 ± 46.2 | -15.51 |
|  | 7^o^C | 87.9 ± 15.1 | -71.96 | 124.6 ± 56.0 | 20.04 |
|  | 18^o^C | 83 ± 15.4 | -73.52 | NR; 7  660 ± 60 | 535.84 |
