# Supplementary material for: Anaesthetic efficacy of Aqui-S, Benzoak, and MS-222 on lumpfish (Cyclopterus lumpus) fries. Impact from temperature, salinity, and fasting
Source: PLoS One. 2019 Jan 22;14(1):e0211080. doi: 10.1371/journal.pone.0211080 (PMC6342319; doi:10.1371/journal.pone.0211080)
Supplement: S2 File — (DOCX) [file pone.0211080.s002.docx]

Table S4. Statistical analysis to figure 1, Induction time.

| **Statistical test** | **Anaesthetic chemical** | **Induction time** | | | | | | | **Significance** |
| --- | --- | --- | --- | --- | --- | --- | --- | --- | --- |
|  |  | **Concentration (mg/L)** | | | | | | |  |
|  |  | **6** | **12,5** | **18** | **25** | **50** |  |  |  |
| Kruskal-Wallis,  Dunn´s Method | **Aqui-S** | NI; 10 | a  NI; 1 | ab | bc | c |  |  | H_3_ = 33.791  (P = < 0.001) |
|  |  | **12,5** | **25** | **37,5** | **50** | **100** |  |  |  |
| Kruskal-Wallis,  Dunn´s Method | **Benzoak** | NI; 10 | a  NI; 2 | ab | bc | c |  |  | H_3_ = 33.019  (P = < 0.001) |
|  |  | **25** | **38** | **44** | **60** | **75** | **100** | **150** |  |
| Kruskal-Wallis,  Dunn´s Method | **MS-222** | NI; 10 | NI; 10 | a  NI; 8 | b | c | cd | d | H_6_ = 69.919  (P = < 0.001) |

NI = Not induced (number of fish)

Table S5. Statistical analysis to figure 2, Recovery time.

| **Statistical test** | **Anaesthetic chemical** | **Recovery time** | | | **Significance** |
| --- | --- | --- | --- | --- | --- |
|  |  | **Exposure time (minutes)** | | |  |
|  |  | **5** | **10** | **20** |  |
| Kruskal-Wallis,  Dunn´s Method | **Aqui-S (18 mg/L)** | a | b | NR; 10 | H_1_ = 10.149  (P = 0.001) |
| ANOVA,  Tukey Test | **Benzoak**  **(37,5 mg/L)** | a | a | b | F = 8.678  (P = 0.001) |
| ANOVA,  Tukey Test | **MS-222**  **(60 mg/L)** | a | b | b | F = 24.270  (P = < 0.001) |

NR = Not Recovered (number of fish)

Table S6. Statistical analysis to figure 3, Induction time at various conditions

| **Statistical test** | **Anaesthetic chemical** | **Induction time** | | | | | **Significance** |
| --- | --- | --- | --- | --- | --- | --- | --- |
|  |  | **Condition** | | | | |  |
|  |  | **Acclimated**  **(Seawater, 12 ^o^C)** | **Brackish water** | **Fasted** | **7^o^C** | **18^o^C** |  |
| Kruskal-Wallis,  Tukey Test | **Aqui-S (18 mg/L)** |  |  |  |  |  | H_4_ = 24.183  (P < 0.001) |
| Kruskal-Wallis,  Tukey Test | **Benzoak**  **(37,5 mg/L)** |  |  |  | *  (P = 0.002) |  | H_4_ = 41.330  (P < 0.001) |
| Kruskal-Wallis,  Dunn´s Method | **MS-222**  **(60 mg/L)** |  |  |  | *  (P < 0.001) | *  (P < 0.001) | H_4_ = 39.580  (P < 0.001) |

* = Statistically significant different from acclimated condition.

Table S7. Statistical analysis to figure 4, Recovery time at various conditions (after 20 minutes exposure)

| **Statistical test** | **Anaesthetic chemical** | **Recovery time** | | | | | **Significance** |
| --- | --- | --- | --- | --- | --- | --- | --- |
|  |  | **Condition** | | | | |  |
|  |  | **Acclimated**  **(Seawater, 12 ^o^C)** | **Brackish water** | **Fasted** | **7^o^C** | **18^o^C** |  |
|  | **Aqui-S (18 mg/L)** | NR; 10 | NR; 8 | NR; 3 | NR; 10 | NR; 10 |  |
| Kruskal-Wallis,  Tukey Test | **Benzoak**  **(37,5 mg/L)** |  |  |  | *  (P = 0.003) | NR; 10 | H_3_ = 26.710  (P < 0.001) |
| Kruskal-Wallis,  Dunn´s Method | **MS-222**  **(60 mg/L)** |  | *  (P = 0.045) |  |  | NR; 7 | H_4_ = 21.397  (P < 0.001) |

* = Statistically significant different from acclimated condition.
